# Antibodies to Peptides in Semiconserved Domains of RIFINs and STEVORs Correlate with Malaria Exposure

**DOI:** 10.1128/mSphere.00097-19

**Published:** 2019-03-20

**Authors:** Albert E. Zhou, Andrea A. Berry, Jason A. Bailey, Andrew Pike, Antoine Dara, Sonia Agrawal, Emily M. Stucke, Amed Ouattara, Drissa Coulibaly, Kirsten E. Lyke, Matthew B. Laurens, Matthew Adams, Shannon Takala-Harrison, Jozelyn Pablo, Algis Jasinskas, Rie Nakajima, Amadou Niangaly, Bourema Kouriba, Abdoulaye K. Kone, J. Alexandra Rowe, Ogobara K. Doumbo, Mahamadou A. Thera, Jigar J. Patel, John C. Tan, Philip L. Felgner, Christopher V. Plowe, Mark A. Travassos

**Affiliations:** a Malaria Research Program, Center for Vaccine Development and Global Health, University of Maryland School of Medicine, Baltimore, Maryland, USA; b The EMMES Corporation, Rockville, Maryland, USA; c Malaria Research and Training Center, University of Sciences, Techniques and Technologies, Bamako, Mali; d Division of Infectious Diseases, Department of Medicine, University of California, Irvine, Irvine, California, USA; e Centre for Immunity, Infection and Evolution, Institute of Immunology and Infection Research, School of Biological Sciences, University of Edinburgh, Edinburgh, United Kingdom; f Roche NimbleGen, Inc., Madison, Wisconsin, USA; g Duke Global Health Institute, Duke University, Durham, North Carolina, USA; University at Buffalo

**Keywords:** *Plasmodium falciparum*, RIFIN, STEVOR, malaria, microarrays, peptide, semiconserved domain, severe malaria, variant surface antigen

## Abstract

The repetitive interspersed family (RIFIN) and the subtelomeric variable open reading frame (STEVOR) family represent two of three major Plasmodium falciparum variant surface antigen families involved in malaria pathogenesis and immune evasion and are potential targets in the development of natural immunity. Protein and peptide microarrays populated with RIFINs and STEVORs associated with severe malaria vulnerability in Malian children were probed with adult and pediatric sera to identify epitopes that reflect malaria exposure. Adult sera recognized and reacted with greater intensity to all STEVOR proteins than pediatric sera did. Serorecognition of and seroreactivity to peptides within the semiconserved domain of STEVORs increased with age and seasonal malaria exposure, while serorecognition and seroreactivity increased for the semiconserved and second hypervariable domains of RIFINs only with age. Serologic responses to RIFIN and STEVOR peptides within the semiconserved domains may play a role in natural immunity to severe malaria.

**IMPORTANCE** Malaria, an infectious disease caused by the parasite Plasmodium falciparum, causes nearly 435,000 deaths annually worldwide. RIFINs and STEVORs are two variant surface antigen families that are involved in malaria pathogenesis and immune evasion. Recent work has shown that a lack of humoral immunity to these proteins is associated with severe malaria vulnerability in Malian children. This is the first study to have compared serologic responses of children and adults to RIFINs and STEVORs in settings of malaria endemicity and to examine such serologic responses before and after a clinical malaria episode. Using microarrays, we determined that the semiconserved domains in these two parasite variant surface antigen families harbor peptides whose seroreactivity reflects malaria exposure. A similar approach has the potential to illuminate the role of variant surface antigens in the development of natural immunity to clinical malaria. Potential vaccines for severe malaria should include consideration of peptides within the semiconserved domains of RIFINs and STEVORs.

## Abstract (precis)

Malaria, an infectious disease caused by the parasite Plasmodium falciparum, causes nearly 435,000 deaths annually worldwide. RIFINs and STEVORs are two variant surface antigen families that are involved in malaria pathogenesis and immune evasion. Recent work has shown that a lack of humoral immunity to these proteins is associated with severe malaria vulnerability in Malian children. This is the first study to have compared serologic responses of children and adults to RIFINs and STEVORs in settings of malaria endemicity and to examine such serologic responses before and after a clinical malaria episode. Using microarrays, we determined that the semiconserved domains in these two parasite variant surface antigen families harbor peptides whose seroreactivity reflects malaria exposure. A similar approach has the potential to illuminate the role of variant surface antigens in the development of natural immunity to clinical malaria. Potential vaccines for severe malaria should include consideration of peptides within the semiconserved domains of RIFINs and STEVORs.

## INTRODUCTION

Malaria in humans is primarily caused by Plasmodium falciparum, accounting for 435,000 annual deaths worldwide in 2017 (1). Repetitive interspersed family (RIFIN) and subtelomeric variable open reading frame (STEVOR) proteins are two P. falciparum variant surface antigen (VSA) families involved in malaria pathogenesis and immune evasion (2–8). Individuals in regions where malaria is endemic likely acquire natural immunity to clinical disease due to production of antibodies against certain VSAs (8–12), but little is known about the relative contributions of antibodies against RIFINs and STEVORs for protection against malaria disease (reviewed in references 13–15).

**FIG 1:**
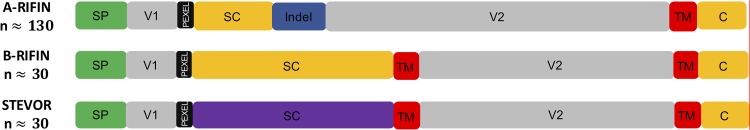
General structure of RIFINs and STEVORs. Protein domains are illustrated as green (signal peptide [SP]), gray (short hypervariable domain [V1] and hypervariable domain [V2]), red (transmembrane domains [TM]), black (PEXEL motif), blue (25-amino-acid sequence found only in RIFIN-A antigens), or orange/purple (semiconserved [SC] or conserved [C] domain). RIFINs are encoded by 160 *rif* genes in the 3D7 reference genome, separated into two subtypes, RIFIN-A and RIFIN-B, depending on sequence, number of transmembrane domains, and subcellular localization. Thirty 3D7 genes encode STEVORs, which are structurally similar to RIFIN-Bs.

P. falciparum reference genome 3D7 includes 160 *rif* genes and 30 *stevor* genes encoding RIFIN and STEVOR proteins, respectively. Both gene families exhibit high sequence diversity (16–18). These proteins are exported to the infected red blood cell surface where portions become exposed to the immune system. RIFINs and STEVORs feature a domain architecture consisting of a signal sequence, a short variable domain (V1), a PEXEL motif, a semiconserved (SC) domain, a second hypervariable domain (V2), and a transmembrane domain inserted into the erythrocyte membrane that precedes a conserved C terminus (Fig. 1). RIFINs are divided into two subfamilies, RIFIN-A and RIFIN-B, with the latter group localized subcellularly (6). RIFIN-As have a unique 25-amino-acid insertion sequence within the SC domain and, typically, a single transmembrane domain (16, 18–20). A number of STEVORs have a second transmembrane domain, similar to RIFIN-Bs (13).

Particular RIFINs may serve as important targets in the development of natural immunity to severe malaria (3, 8, 21, 22). Increased antibody titers corresponding to four recombinant RIFINs correlated with suppression of malaria symptoms and rapid parasite clearance in asymptomatic Gabonese children (23). Anti-RIFIN antibodies may limit disease severity by disrupting rosette formation (24), promoting phagocytosis of infected red blood cells (21), and preventing RIFIN binding to host leukocyte immunoglobulin-like receptor B1 (LILRB1) (3). Such RIFIN binding to LILRB1 receptors on B and NK immune cells limits the host immune response, potentially increasing susceptibility to severe malaria.

We are only beginning to understand natural immunity to STEVOR proteins. Antibodies to STEVORs weakly inhibit merozoite adhesion and invasion *in vitro* (25, 26). Given the structural similarity of STEVORs and RIFINs (18), it is possible that antibodies to STEVORs may play a role in clinical protection.

We recently identified a subset of RIFINs and STEVORs associated with severe malaria vulnerability in Malian children (22). Sera from children with severe malaria exhibited gaps in immunity, designated “lacunae,” to three RIFINs and three STEVORs compared with matched sera from children with uncomplicated malaria infections. Given these findings, we further examined the serologic response to this subset of RIFINs and STEVORs in Malian children and adults. Our goal was to identify the RIFIN and STEVOR domains and amino acid residues that contain epitopes reflecting malaria exposure. We hypothesized that antibodies against RIFINs and STEVORs would target more epitopes within the SC domain than within the V1 and V2 domains, given the SC domain’s membrane orientation and relative sequence homology (18, 27, 28).

Protein and peptide microarrays are tools that permit analyses of immune responses to large numbers of malaria proteins and constituent amino acid residues (29, 30). Protein microarrays measure levels of antibody binding to tertiary protein structures, while peptide arrays provide increased resolution to detect individual epitopes. We populated protein and peptide microarrays with these three RIFINs and three STEVORs associated with severe malaria vulnerability as well as with three additional STEVORs and probed them with sera from Malian adults and children to identify epitopes that are serorecognized and seroreactive after naturally acquired malaria infection.

(This work was presented in part at the 66th annual meeting of the American Society of Tropical Medicine and Hygiene, Baltimore, MD [31], and at the 67th annual meeting of the American Society of Tropical Medicine and Hygiene in New Orleans, LA, October 2018 [32].)

## RESULTS

### Malian adults had greater seroreactivity to STEVOR proteins than Malian children.

Sera from Malian adults recognized all three RIFINs and six STEVORs on the protein microarray, while sera from Malian children recognized all three RIFINs but only four of the six STEVORs (P. falciparum 3D7_1300900 [PF3D7_1300900], PF3D7_0300400, PF3D7_0115400, and PF3D7_1254100) (Table 1), including two associated with cerebral malaria vulnerability (PF3D7_0300400 and PF3D7_0115400) (22). The two STEVORs that were not recognized by pediatric sera were PF3D7_0832000 and PF3D7_0832600. The former is associated with vulnerability to cerebral malaria as well as severe malarial anemia (22).

**TABLE 1:** Sera from Malian adults recognized a greater number of STEVORs than pediatric sera

Surface antigen	Serorecognition^*a*^	
	Adults (*n* = 18)	Children (*n* = 75)
RIFINs		
PF3D7_0732400	✓	✓
PF3D7_1041100	✓	✓
PF3D7_0732400	✓	✓

STEVORs		
PF3D7_1300900	✓	✓
PF3D7_0832000	✓	X
PF3D7_0832600	✓	X
PF3D7_0300400	✓	✓
PF3D7_0115400	✓	✓
PF3D7_1254100	✓	✓

a ✓, serorecognition; X, lack of serorecognition.

**FIG 2:**
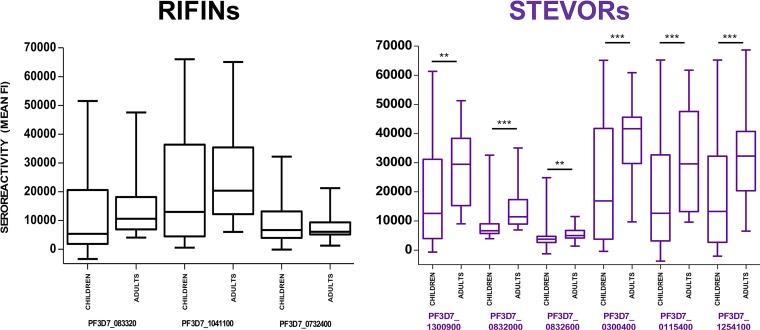
Protein microarray seroreactivity of three RIFINs (black) and six STEVORs (purple). Malian adult sera exhibited greater seroreactivity to all six STEVORs than pediatric sera. There were no significant differences between adult and pediatric seroreactivity to the three RIFINs. Data representing fluorescence intensity (FI) for sera from adults (*n* = 18) and children (*n* = 75) were compared using a Mann-Whitney test (**, *P* < 0.01; *** *P* < 0.001). The *y* axis shows seroreactivity represented by mean fluorescence intensity. Each box plot shows the median, bracketed by the lower 25th and upper 75th percentiles, and the minimum and maximum values.

Sera from Malian adults reacted more intensely to all six STEVORs than the pediatric sera did. In contrast, the levels of RIFIN seroreactivity were comparable between the two groups, with no significant differences (Fig. 2).

### “Serorecognition” of and seroreactivity to peptides within the semiconserved domains and second hypervariable (V2) domains of all three RIFINs reflect age-related differences in malaria exposure.

**FIG 3:**
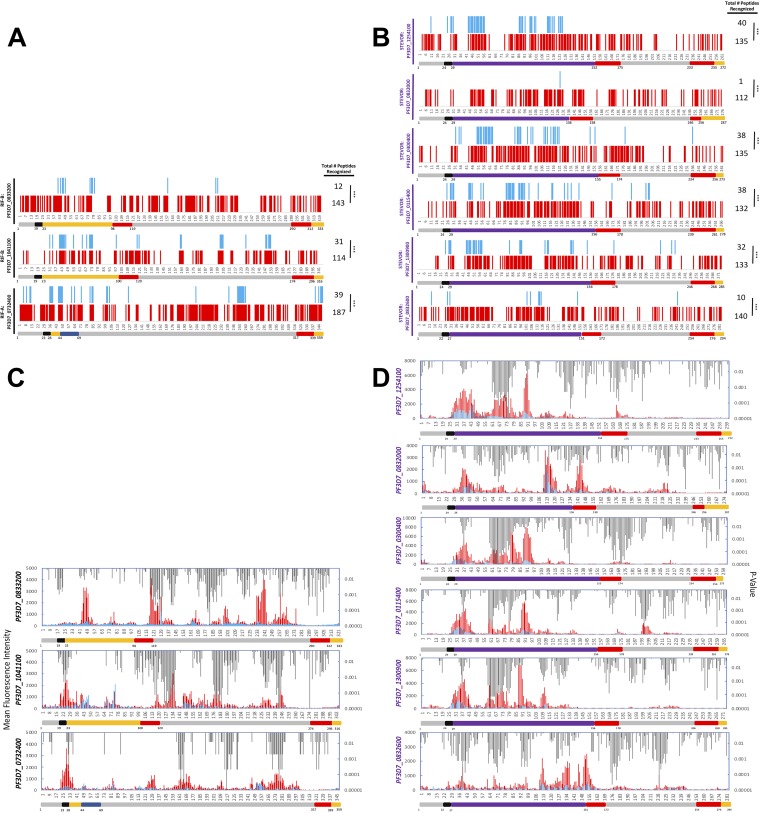
Malaria-exposed adults (*n* = 10) demonstrated greater serorecognition of and seroreactivity to peptides of three RIFINs (A and C) and six STEVORs (B and D) than children (*n* = 10) as measured using peptide microarrays. (A and B) Malian adult sera (red) recognized significantly more (A) RIFIN and (B) STEVOR total peptides than preseason pediatric sera (blue). The horizontal axis depicts the relative location of the N-terminal amino acid for each peptide. ***, significant difference between adults and children in serorecognized peptide counts (*P* < 0.05; McNemar’s test). (C and D) Malian adult sera (red) had increased seroreactivity to subsets of (C) RIFIN and (D) STEVOR peptides compared with pediatric sera (blue) (*P* values are indicated in gray on the secondary *y* axis; Wilcoxon rank-sum test).

For each of the three RIFINs, Malian adult sera recognized significantly more total peptides than the pediatric sera did (Fig. 3A). For all three RIFINs, Malian adults had greater counts of serorecognized peptides in the SC and V2 domains than Malian children did, whereas in the V1 domain, this was true for two of the three RIFINs (Table 2). In contrast to the protein microarray results, the peptide array data showed differential levels of RIFIN seroreactivity between Malian adults and children. Adult sera had greater seroreactivity to several subsets of RIFIN peptides in the V1, SC, and V2 domains than the pediatric sera did (Fig. 3C).

**TABLE 2:** Summary of peptide serorecognition comparisons for the domains of three RIFINs and six STEVORs

Surface antigen and serum group	No. of peptides recognized (*P* value)^*a*^			
	Total	V1	SC	V2
RIFIN				
Adults vs Pediatric				
PF3D7_0833200	143 vs 12***	19 vs 0**	35 vs 9***	73 vs 3***
PF3D7_1041100	114 vs 31***	3 vs 0 (0.617)	32 vs 17***	55 vs 13***
PF3D7_0732400	187 vs 39***	15 vs 4**	26 vs 16**	120 vs 19***
Pediatric Infected—day 90 vs day 0				
PF3D7_0833200	99 vs 27***	15 vs 3 (0.480)	39 vs 13 (0.134)	28 vs 9***
PF3D7_1041100	88 vs 50**	5 vs 5 (1)	37 vs 23 (0.480)	34 vs 18 (0.077)
PF3D7_0732400	139 vs 67***	12 vs 8 (0.131)	30 vs 16***	77 vs 34**
				
STEVORs				
Adults vs Pediatric				
PF3D7_1254100	135 vs 40***	13 vs 1**	80 vs 26***	17 vs 0***
PF3D7_0832000	112 vs 1***	7 vs 0*	54 vs 1***	27 vs 0***
PF3D7_0300400	135 vs 38***	5 vs 0 (0.134)	85 vs 34***	22 vs 1***
PF3D7_0115400	132 vs 38***	4 vs 0 (0.371)	81 vs 31***	24 vs 4***
PF3D7_1300900	133 vs 32***	3 vs 0 (0.617)	80 vs 26***	22 vs 3***
PF3D7_0832600	140 vs 10***	9 vs 1*	80 vs 8***	29 vs 1***
Pediatric infected—day 90 vs day 0				
PF3D7_1254100	106 vs 62***	13 vs 4 (0.248)	71 vs 49**	8 vs 2 (1)
PF3D7_0832000	64 vs 13***	1 vs 0 (1)	32 vs 9***	16 vs 4 (0.617)
PF3D7_0300400	105 vs 62***	8 vs 1 (0.617)	67 vs 54**	15 vs 3 (0.134)
PF3D7_0115400	107 vs 59***	3 vs 0 (1)	77 vs 28**	13 vs 9 (1)
PF3D7_1300900	115 vs 58***	7 vs 0 (1)	79 vs 45***	13 vs 6*
PF3D7_0832600	82 vs 23***	9 vs 1 (0.134)	56 vs 18 ***	5 vs 2 (0.480)

a *, *P* < 0.01; **, *P* < 0.001; ***, *P* < 0.0001.

**FIG 4:**
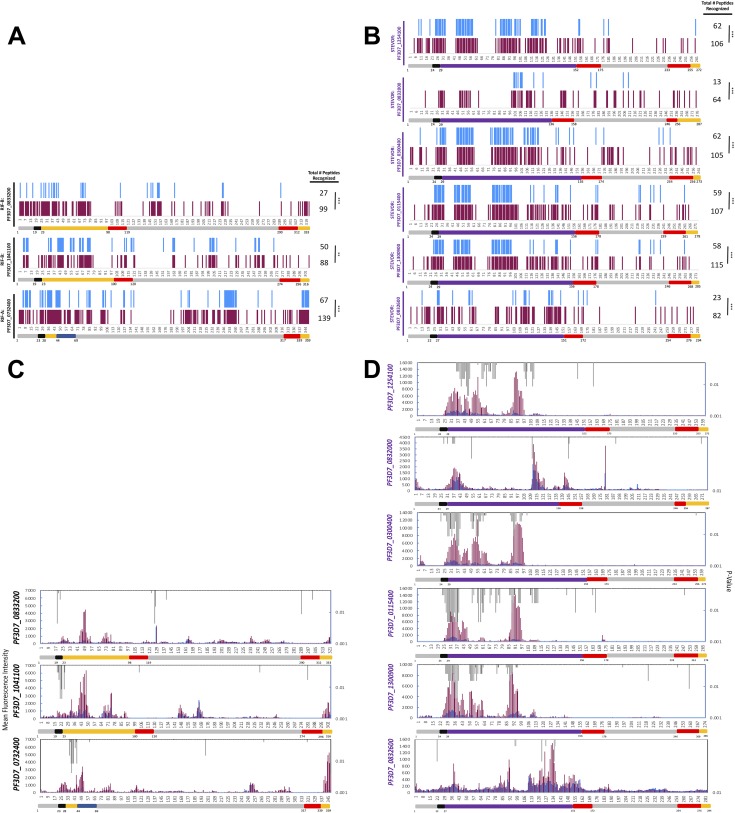
Children with recent clinical malaria episodes (*n* = 6) exhibited increased serological responses to three RIFINs (A and C) and six STEVORs (B and D) during the transmission season as measured using peptide microarrays. (A and B) Day 90 pediatric sera (maroon) recognized significantly more (A) RIFIN and (B) STEVOR peptides in total than the same day 0 pediatric sera from before the malaria season (blue). The *x* axis depicts the peptide number corresponding to the sequence from the N terminus to the C terminus for each respective antigen. ***, significant difference in serorecognized peptide counts between day 0 and day 90 (*P* < 0.05; McNemar’s test). (C and D) Day 90 sera (maroon) had increased seroreactivity to subsets of (C) RIFIN and (D) STEVOR peptides compared with matched sera before the malaria transmission season (blue) (*P* values in gray; Wilcoxon signed-rank test).

Sera collected at day 90 during the transmission season from children who had already experienced at least one clinical malaria episode showed serorecognition of significantly more total peptides for each of the three RIFIN proteins than paired preseason pediatric sera (Fig. 4A). These seasonal differences were not consistently significant for the V1, SC, or V2 RIFIN domains alone (Table 2). Day 90 pediatric sera did not consistently react more intensely than day 0 preseason sera to any group of peptides within a particular RIFIN domain (Fig. 4C). Similar patterns were present in sera from all children (see Fig. S1 in the supplemental material). In contrast, samples collected from children who did not experience a malaria episode during the malaria transmission season did not differ from preseason sera in counts of recognized peptides for any of the three RIFIN proteins (*n* = 4; Fig. S2).

10.1128/mSphere.00097-19.1 FIG S1 Peptide microarray serorecognition of and reactivity to three RIFINs and six STEVORs using matched pediatric sera before and during the malaria transmission season (*n* = 10). (A) Pediatric sera at day 90 (bottom, gold) recognized significantly more RIFIN and STEVOR peptides than pediatric sera from before the malaria season (top, sky blue). (B) Sera from day 90 of the malaria transmission season (gold) differed in seroreactivity for only a few peptides compared to sera at the start of the season (sky blue) (*P* values in gray; Wilcoxon-signed rank test). Download FIG S1, TIF file, 2.7 MB. Copyright © 2019 Zhou et al. 2019 Zhou et al. This content is distributed under the terms of the Creative Commons Attribution 4.0 International license.

10.1128/mSphere.00097-19.2 FIG S2 Peptide microarray serorecognition and reactivity of matched pediatric sera of children who did not experience a clinical malaria episode during the malaria transmission season (*n* = 4). (A) Day 90 pediatric sera that did not experience a clinical malaria episode (bottom, magenta) were not significantly different from matched sera collected at the start of the malaria season (top, teal). (B) For sera obtained during the malaria transmission season (magenta), seroreactivity differed for only a few peptides compared to the start of the season (teal) (*P* values in gray; Wilcoxon-signed rank test). Download FIG S2, TIF file, 1.4 MB. Copyright © 2019 Zhou et al. 2019 Zhou et al. This content is distributed under the terms of the Creative Commons Attribution 4.0 International license.

### Serorecognition of and seroreactivity to peptides within the semiconserved domains of STEVORs reflect differences in age and seasonal malaria exposure.

For each of the six STEVORs, Malian adult sera recognized significantly more total peptides than pediatric sera. These age-related differences were also present for particular individual STEVOR domains. For each of the six STEVORs, adult sera recognized more peptides in the SC and V2 domains than the pediatric sera (Fig. 3B; see also Table 2). In contrast, children did not differ from adults in counts of recognized V1 domain peptides for four of the six STEVORs. Adult sera also reacted more intensely to subsets of STEVOR peptides within the SC and V2 domains than pediatric sera (Fig. 3C and D).

Pediatric sera collected during the malaria transmission season from children who had already experienced at least one clinical malaria episode recognized significantly more peptides across the full length of each of the six STEVORs than pediatric sera from the same children collected before the start of the transmission season (Fig. 4B). These seasonal differences were significant for individual STEVOR domains: day 90 sera recognized more peptides within the SC domain than preseason sera for each of the six STEVORs, but this was not the case for any of the V1 domains (Table 2). Day 90 pediatric sera reacted more intensely than day 0 sera to subsets of STEVOR peptides within the SC domains of four STEVORs (PF3D7_1300900, PF3D7_0300400, PF3D7_0115400, and PF3D7_1254100) (Fig. 4D). Similar seasonal trends were present in sera from all children (Fig. S1). In contrast, sera collected from children who did not experience a malaria episode during the malaria transmission season did not differ from preseason sera in counts of recognized peptides for any of the six STEVOR proteins (*n* = 4; Fig. S2).

### Semiconserved and second hypervariable (V2) VSA domains harbor peptides reflecting malaria exposure.

**FIG 5:**
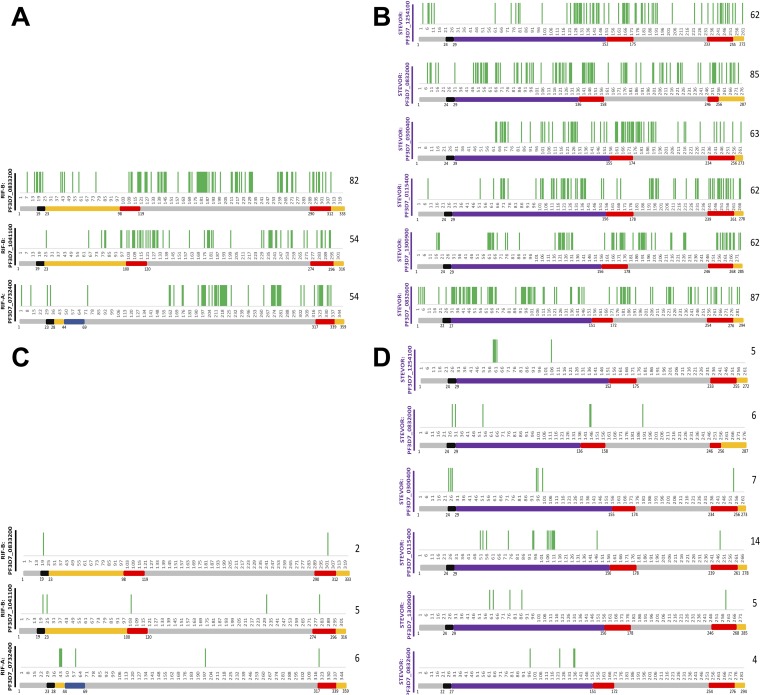
Peptides that were both serorecognized and differentially seroreactive in group comparisons were present in both RIFINs and STEVORs and captured differences in ages and seasonal exposures. These peptides for adult-child comparisons were present in all (A) three RIFINs and (B) six STEVORs, including within the second variable (V2) domain for RIFINs and in both the semiconserved (SC) and second variable (V2) domain for STEVORs. These peptides corresponding to matched pediatric seasonal comparisons were sparse and not uniformly located within the three RIFINs (C), whereas the SC domain consistently harbored such peptides in the STEVORs (D). The number of these peptides is listed to the right of each protein designation.

We identified peptides that were both serorecognized and differentially seroreactive in group comparisons. These peptides were present throughout all three RIFIN and six STEVOR proteins (Fig. 5; see also Table S1 in the supplemental material). Such peptides differentiating sera of children from sera of adults were consistently located in the RIFIN V2 domain and in both the SC and V2 STEVOR domains (Fig. 5A and B). Similarly, these peptides differentiating sera collected before the malaria transmission season from sera collected during the malaria transmission season for children experiencing clinical malaria episodes were sparse and not uniformly located within any of the three RIFINs, whereas the semiconserved domain consistently harbored such STEVOR peptides (Fig. 5C and D).

10.1128/mSphere.00097-19.3 TABLE S1 RIFIN and STEVOR protein and peptide sequences. Download Table S1, XLSX file, 0.09 MB. Copyright © 2019 Zhou et al. 2019 Zhou et al. This content is distributed under the terms of the Creative Commons Attribution 4.0 International license.

## DISCUSSION

We previously identified three RIFINs and three STEVORs associated with severe malaria vulnerability in Malian children (22). Here, we determined that the semiconserved domains of these RIFINs and STEVORs were hot spots for peptides associated with malaria exposure. These peptides likely include epitopes and may contain critical amino acid residues associated with malaria exposure and, potentially, with immunity to clinical malaria.

Protein microarray data demonstrated age-related differences in serologic responses to RIFINs and STEVORs. Serorecognition of the three RIFINs and six STEVORs differentiated between Malian adults and malaria-naive North American controls but not consistently between Malian children and North American controls. Children had signatures of serorecognition to all three RIFINs but to only four of the six STEVORs, indicating that serorecognition of RIFINs may be acquired early in life, while serorecognition of STEVORs may be acquired more gradually. Protein seroreactivity comparisons reflected similar age-related differences. Malian adult sera reacted more intensely than pediatric sera to all six STEVORs but not differentially to any of the three RIFINs.

Peptide microarray data revealed age-related differences in seroreactivity and allowed localization of the discriminatory antibody responses to particular regions within each protein. Adult sera recognized significantly more peptides in the semiconserved and V2 domains of all three RIFINs and six STEVORs than pediatric sera. Likewise, adult sera had more reactivity than pediatric sera to subsets of peptides across each of the nine antigens. Only serorecognition of peptides within the STEVOR semiconserved domain and increased reactivity to peptides within the semiconserved domains of four STEVORs consistently differentiated between pediatric sera obtained at the beginning of the malaria transmission season and the sera obtained during the malaria transmission season. There was increased serorecognition of and seroreactivity to semiconserved domain STEVOR peptides during pediatric malaria infections, while seroreactivity to RIFIN peptides within particular domains was more variable.

Our results suggest that the semiconserved domain of RIFINs and STEVORs is exposed on the erythrocyte surface, given the predominance of peptides that were both serorecognized and differentially seroreactive in group comparisons within this region (Fig. 5). RIFIN and STEVOR hypervariable domains were originally believed to be the only portions exposed on the erythrocyte surface, while the semiconserved domains remained located within (16, 18). Recent work suggested a single transmembrane topology for some RIFINs and STEVORs, such that the semiconserved domain would also be exposed on the host cell surface (18), which is consistent with our findings. We previously showed that malaria-exposed sera react to the most conserved sequences of P. falciparum erythrocyte membrane protein 1 (PfEMP1) (33), which, coupled with these results, indicates that conserved sequences may be recognized by the immune system before highly variable regions.

The semiconserved domain has not previously been identified as a target of the human immune response. A recent study used peptide microarrays to identify binding sites of rabbit antibodies directed against RIFINs and STEVORs and localized this reactivity to the C-terminal, intracellular conserved domain (34). A subsequent study used a peptide array to identify regions of a RIFIN-A targeted by rosette-disrupting pediatric sera (24). Surprisingly, antibodies bound not to the extracellular region but instead to the conserved intracellular domain. In contrast to those studies, our current study was the first to compare malaria-exposed serologic responses of children to those of adults, which allowed us to identify the semiconserved domain as a marker of age-related malaria exposure.

We have previously shown that malaria-exposed sera react to the intracellular portion of PfEMP1s (33). Although intracellular regions are likely shielded from antibody recognition, these regions may become exposed to the immune system after erythrocyte lysis. Immune exposure after erythrocyte lysis may also explain the similar levels of seroreactivity of different RIFIN types that we observed. The one RIFIN-A and two RIFIN-Bs probed on both the protein and peptide microarrays did not have distinct serorecognition and seroreactivity patterns in either age-related or seasonal comparisons. RIFIN-As are found on the surface of infected red blood cells and potentially mediate erythrocyte adhesion (20). In contrast, RIFIN-Bs remain within the erythrocyte, helping maintain parasite metabolism (6, 18). The finding of similar seroprofiles of RIFIN-As and RIFIN-Bs despite differences in functions and cellular location may have been a consequence of immune exposure of both RIFIN types after erythrocyte lysis. As such, these serologic responses may reflect malaria exposure but not protective immunity.

Differences between the serologic responses of STEVORs and RIFINs may be linked to differences in their respective functions, which are not fully understood. STEVORs play a role in merozoite invasion and rosetting *in vitro* (25, 26). Our results revealed peptides that were both serorecognized and differentially seroreactive in group comparisons localized within the semiconserved regions of six STEVORs. In a prior study, peptides from these regions of a STEVOR bound native red blood cell surface proteins (35). Sera raised against these STEVOR peptides inhibited *in vitro* invasion processes. Antibodies to peptides that were both serorecognized and differentially seroreactive in group comparisons that we have identified in the semiconserved regions of STEVORs may protect against erythrocyte binding in clinical malaria syndromes and should be evaluated further in *in vitro* studies.

It has previously been hypothesized that children acquire immunity to severe malaria after a single malaria episode (11, 36). We showed a marked increase in serorecognition to peptides of all of the STEVORs and RIFINs examined following a clinical episode of malaria, including the three STEVORs and three RIFINs previously associated with severe malaria vulnerability in Malian children (22). This rapid acquisition of antibodies to epitopes may indicate how severe malaria immunity could develop after a single clinical episode. Interestingly, a study in Ghanaian children found that antibodies to STEVOR PF3D7_0300400 did not protect naive Ghanaian children from subsequent parasitemia; these antibodies served as a marker of infection but were not associated with protective immunity (28). An immune response to a single STEVOR may not confer protection against severe malaria, but a broad response to a larger set of variant surface antigens may be a better predictor of protection against clinical disease. Further microarray studies of a more comprehensive set of variant surface antigens using sera from children with severe malaria may provide crucial insights into specific targets of natural immunity.

This study had some limitations. Antigens on the protein microarray were fabricated with a cell-free Escherichia coli-based transcription and translation system, which may not represent the actual conformations of wild-type RIFINs or STEVORs. However, this protein array platform has produced antibody responses to malaria vaccine candidate antigens that closely correlate with measurements of purified versions of these proteins using enzyme-linked immunosorbent assays (ELISAs) (37). Findings of studies performed with protein microarrays parallel predicted serological responses in malaria-exposed children and adults (22, 33, 37, 38). Similarly, the short linear peptides used on the peptide microarray may fail to capture conformational or discontinuous epitopes that are important to antibody responses. Yet the majority of antibodies bind to short amino acid sequences (39), and the 16-amino-acid peptides demonstrate some secondary structure, suggesting that conformational epitopes may still be present. While we successfully detected signatures of exposure, some epitopes may have been missed given the limited number of pediatric samples assayed. Finally, the RIFINs and STEVORs included on the two microarrays comprise only a small subset of the many variants found in the parasite. Our platforms may not include critical immunological variants of these variant surface antigens.

Protein and peptide microarrays populated with a panel of RIFINs and STEVORs associated with severe malaria vulnerability revealed that the semiconserved domains of both RIFINs and STEVORs serve as markers of malaria exposure. To comprehensively identify epitopes associated with severe malaria vulnerability and whether differences in exposure are truly correlated with protection, protein and peptide microarrays populated with a comprehensive set of RIFINs and STEVORs should be probed with sera from severe malaria case-control studies. Further analyses and characterization of relevant STEVORs and RIFINs through invasion inhibition, rosetting, and phagocytosis assays will provide insight into the role each protein plays in the development of severe malaria.

## MATERIALS AND METHODS

### Microarray construction and controls.

Both the protein and peptide microarrays included three full-length RIFIN antigens (PF3D7_0833200 [RIFIN-B], PF3D7_104110 [RIFIN-B], and PF3D7_0732400 [RIFIN-A]) and three full-length STEVOR antigens (PF3D7_0832000, PF3D7_0300400, and PF3D7_0115400) associated with cerebral malaria vulnerability; PF3D7_0832000 is also associated with vulnerability to severe malarial anemia (22). We also included three additional 3D7 STEVORs (PF3D7_1300900, PF3D7_0832600, and PF3D7_1254100) that were used to evaluate antibody responses in Ghanaian children and adults (28). The signal peptide sequence was omitted from all proteins.

Protein microarray construction followed a four-step process: (i) PCR amplification of each complete or partial P. falciparum open reading frame, (ii) recombination cloning, (iii) *in vitro* transcription/translation (IVTT), and (iv) microarray chip printing (29). This IVTT protein microarray platform has produced antibody measurements that correlate with those of purified proteins of several malaria vaccine candidate antigens on ELISAs (37). The peptide array represents protein sequences of interest with a peptide tiling design incorporating peptides that are 16 amino acids in length, where the start location of peptide sequences is offset by 4 amino acids (i.e., a 12-amino-acid sequence overlap between contiguous peptides). After the design was finalized, peptide arrays were synthesized through light-directed solid-phase peptide synthesis as previously described (30). Serum samples were bound to peptide arrays and labeled with an anti-human IgG secondary antibody with an Alexa Fluor 647 fluorescent label.

The amino acid positions of the start and finish of RIFIN and STEVOR domains for each antigen were defined using the PEXEL motif and transmembrane domains as anchors for bioinformatic alignment, based on previous analyses and the PlasmoDB database (6, 7, 16, 20, 34, 40).

### Study population.

Arrays were probed with sera of individuals from Bandiagara, a town of 13,634 inhabitants (2002 census) in the Dogon region in east-central Mali. P. falciparum infections represent 97% of the malaria infections in this region. Malaria transmission is sharply seasonal, with minimal transmission at the height of the dry season in March; fewer than one infected mosquito bite per person per month at the start and end of the transmission season in June and December, respectively; and a peak of up to 60 infected bites per person per month in September (41). Children experience one to two clinical malaria episodes per season (42). The average age of a child with severe malaria is 38.7 months (43), and the mean age of patients diagnosed with uncomplicated malaria is 10 years (44).

We probed protein microarrays with sera from Malian adults aged 18 to 55 years enrolled in the control arm of a phase I AMA1 vaccine (FMP2.1/AS02A) trial (*n* = 18) (48) and sera from Malian children aged 1 to 6 years enrolled in the control arm of a phase II AMA1 vaccine (FMP2.1/AS02A) trial (*n* = 75) (45, 46). These serum samples were collected at the start of and during the malaria transmission season. These control volunteers did not receive an intervention affecting their risk for malaria infection, allowing study of natural immunity. Sera from 10 malaria-naive North American blood donors served as negative controls.

A high-density peptide microarray was probed with sera from a subset of randomly selected individuals whose sera was used for the protein microarray, including sera from 10 Malian adults and 10 Malian children and 5 malaria-naive North American control samples. Pediatric sera included time points before (day 0) and during (day 90) the malaria transmission season. Among the 10 children, 6 subjects experienced at least one clinical malaria episode before the day 90 time point, defined as having malaria symptoms and P. falciparum parasites on a blood smear. The average age for these six children at enrollment was 3.1 ± 0.5 years (mean ± standard error), compared to 4.3 ± 0.2 years for the remaining four children (*P* = 0.06, Wilcoxon rank-sum test).

### Ethics statement.

The protocols were approved by institutional review boards of the University of Bamako Faculty of Medicine; the University of Maryland, Baltimore; and the U.S. Army Surgeon General. Written informed consent was obtained before screening and enrollment in the trials. Verbal consent of illiterate parents or guardians was provided and documented using thumbprints and verified by independent witnesses.

### Statistical analysis.

We applied data analysis techniques previously used to evaluate serologic responses with P. falciparum protein microarrays (22, 37, 47). For protein microarrays, fluorescence intensity was defined as the raw signal intensity value reduced by the mean for the no-DNA negative controls to adjust for differences in seroreactivity to the IVTT reaction buffers and cell-free E. coli system. Given the synthetic fabrication process for peptide microarrays, no adjustment for background fluorescence intensity was necessary; raw fluorescence intensity represented peptide seroreactivity. Group serorecognition of a protein or peptide fragment was defined as detection of a level of fluorescence intensity significantly greater than that of the malaria-naive North American control group, based on a two-sample Kolmogorov-Smirnov test. Serorecognition differences between two populations for the peptide array were calculated with a two-tailed McNemar’s test. Levels of seroreactivity of a protein fragment or peptide were compared among groups using a nonparametric Wilcoxon rank sum test for unmatched group-wise comparisons. Levels of seroreactivity for paired samples across time points were compared using a Wilcoxon signed-rank test. Finally, we identified peptides that not only were serorecognized by a single population but also had differentially increased seroreactivity in a given comparison. All *P* values presented represent two-sided values determined without correcting for multiple comparisons, as was previously done in other microarray analyses (22, 24, 38). Statistical analyses were computed using either R Project for Statistical Computing (version 3.4.3) or GraphPad (version 5.04).
