# Supplementary figures and images for: Antibodies to Peptides in Semiconserved Domains of RIFINs and STEVORs Correlate with Malaria Exposure
Source: mSphere. 2019 Mar 20;4(2):e00097-19. doi: 10.1128/mSphere.00097-19 (PMC6429043; doi:10.1128/mSphere.00097-19)

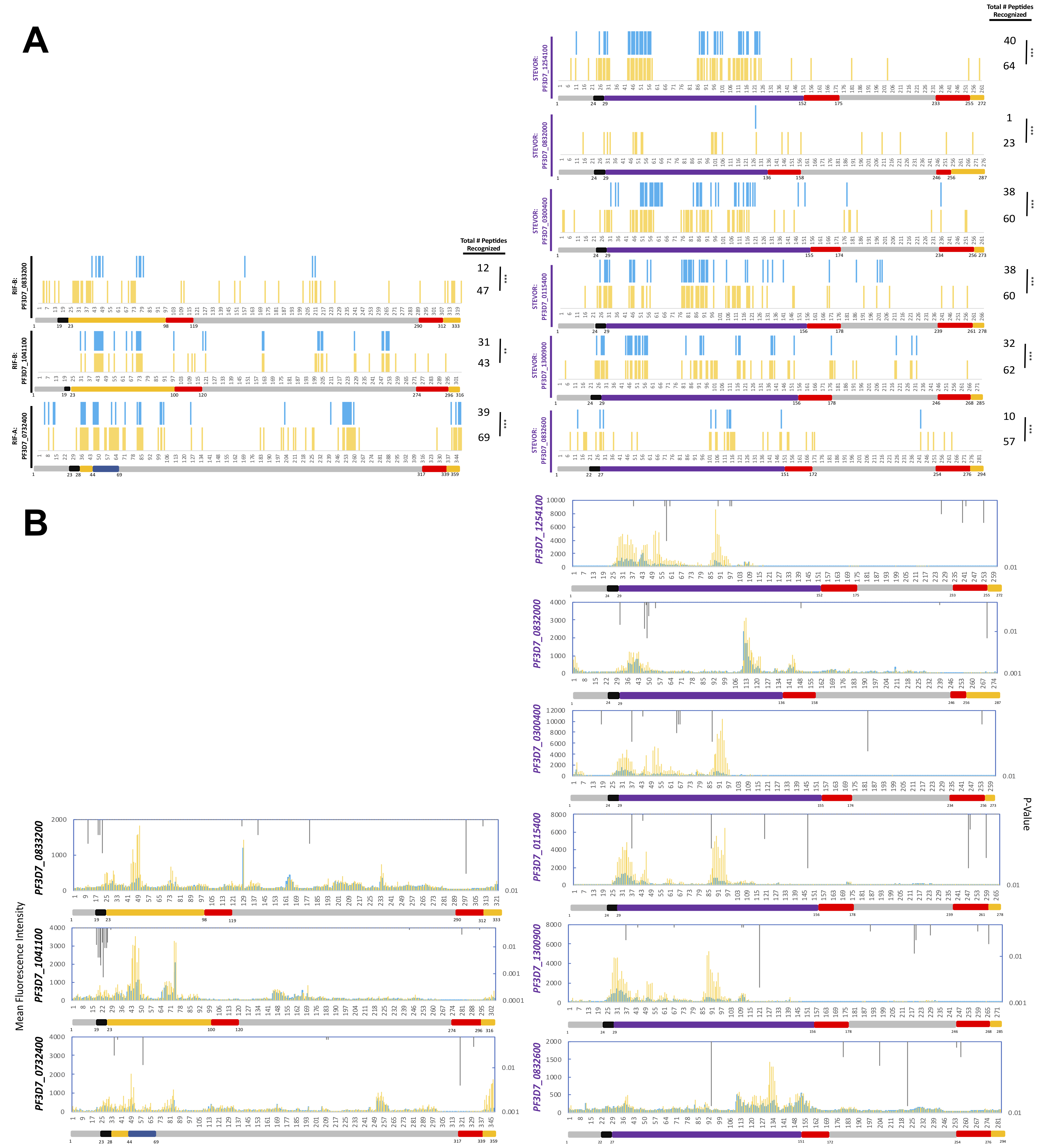

Supplement: FIG S1 [file mSphere.00097-19-sf001.tif]
